# Fruit Fly in a Challenging Environment: Impact of Short-Term Temperature Stress on the Survival, Development, Reproduction, and Trehalose Metabolism of *Bactrocera dorsalis* (Diptera: Tephritidae)

**DOI:** 10.3390/insects13080753

**Published:** 2022-08-22

**Authors:** Chun Yu, Runa Zhao, Wei Zhou, Yingna Pan, Hui Tian, Zhengyan Yin, Wenlong Chen

**Affiliations:** Guizhou Provincial Key Laboratory for Agricultural Pest Management of the Mountainous Region, Institute of Entomology, Scientific Observing and Experimental Station of Crop Pest in Guiyang, Ministry of Agriculture, Guizhou University, Guiyang 550025, China

**Keywords:** growth and development, glycogen, trehalose, temperature tolerance

## Abstract

**Simple Summary:**

*Bactrocera dorsalis* (Hendel) is a widespread and economically important insect pest, infesting various fruits and vegetables. Due to the instability of climate change in early spring and autumn, extreme cold and hot temperatures were developed in a short period of time. Exposure to sudden short-term high or low temperatures may affect the reproduction, development, and physiological changes of *B. dorsalis*. In this study, we determined the effects of short-term temperature treatments on the growth, development, fecundity, and trehalose metabolism of *B. dorsalis*. The results showed that development and reproduction of the flies were negatively affected when temperature was below 10 °C; or more than 31 °C, even causing permanent sterility at extreme temperatures. The changes of glucose, glycogen, trehalose, and trehalose-6-phosphate synthase level had a correlation with the population dynamics of the fruit flies. Our present study can provide a scientific basis for population monitoring, prediction, and comprehensive prevention of the fruit fly.

**Abstract:**

An understanding of physiological damage and population development caused by uncomfortable temperature plays an important role in pest control. In order to clarify the adaptability of different temperatures and physiological response mechanism of *B. dorsalis*, we focused on the adaptation ability of this pest to environmental stress from physiological and ecological viewpoints. In this study, we explored the relationship between population parameters and glucose, glycogen, trehalose, and trehalose-6-phosphate synthase responses to high and low temperatures. Compared with the control group, temperature stress delayed the development duration of all stages, and the survival rates and longevity decreased gradually as temperature decreased to 0 °C and increased to 36 °C. Furthermore, with low temperature decrease from 10 °C to 0 °C, the average fecundity per female increased at 10 °C but decreased later. Reproduction of the species was negatively affected during high-temperature stresses, reaching the lowest value at 36 °C. In addition to significantly affecting biological characteristics, temperature stress influenced physiological changes of *B. dorsalis* in cold and heat tolerance. When temperature deviated significantly from the norm, the levels of substances associated with temperature resistance were altered: glucose, trehalose, and TPS levels increased, but glycogen levels decreased. These results suggest that temperature stresses exert a detrimental effect on the populations’ survival, but the metabolism of trehalose and glycogen may enhance the pest’s temperature resistance.

## 1. Introduction

The oriental fruit fly, *Bactrocera dorsalis* (Hendel), has a wide host range and primarily infests over 250 fruit and vegetable species belonging to 46 families [1,2]. This species establishes a stable population in tropical and subtropical areas and is a destructive pest found in Fujian, Hainan, Guangxi, Guizhou, Yunnan, and other provinces in China, and it has spread rapidly and rampantly in southern and southwestern China [3]. Owing to its wide geographical distribution, host range, and economic impacts, *B. dorsalis* is considered a major threat to global agriculture [4].

Temperature is considered a key factor that influences insect biology and behavior [5,6], affecting them directly by controlling their physiological tolerance limits or indirectly by causing phenological changes and nutrient levels in adjacent environments [7,8]. Several studies have been conducted over the last few decades focusing on the survival and adaptation strategies of ectotherm animals under temperature stress [9]. Although many insects have evolved mechanisms that enable them to respond effectively to normal temperature changes, they continue to be affected by abnormal climate change in nature, which sometimes exceeds their physiological limit and results in the death [10,11]. For instance, a high temperature of 45 °C and a low temperature of −10 °C significantly affected the development and feathering rhythm of *Sarcophaga crassipalpis* [12]. Al-Behadili reported that pupation and adult emergence of *Ceratitis capitata* is significantly reduced after low temperature exposure [13]. In addition, *Frankliniella occidentalis* exhibits altered reproductive adaptation and levels of small molecular compounds such as carbohydrates after exposure to 45 °C [14].

Insects have evolved various strategies to adapt to harsh environmental conditions and respond to external temperature stress through various physiological and metabolic adaptations [15]. For example, heat shock proteins are known to be one important mechanism for resisting the stress caused by an external temperature change [16]. In addition, trehalose is reported to be an important stress metabolite associated with insect tolerance, protects cells from dehydration, drought, heat stress, freezing, and high osmotic pressure [17,18,19]. This sugar can be converted to glucose via hydrolysis, and glucose can be interconverted to glycogen during carbohydrate metabolism in insects. Trehalose contributes to energy metabolism to maintain insects’ life activities such as growth, development, molting, flight, and chitin biosynthesis [20,21]. For example, *Drosophila melanogaster* has a significantly higher level of trehalose, which protects the structure of biological macromolecules from degradation in a dry and water-stressed environment [22]. After 3 h of low-temperature treatment, the trehalose level increased five- or sixfold in *Heterorhabditis bacteriophora* compared with that in the control group [23]. Furthermore, the levels of low-molecular-weight sugars in *Myzus persicae* gradually reduced at repeated high-temperature exposure to resist temperature-related damage [24].

Temperature significantly affects the levels of enzymes and rates of enzymatic reactions [25,26]. Trehalose-6-phosphate synthase (TPS) catalyzes the synthesis of trehalose, which is primarily synthesized in insects’ fat bodies via the TPS/trehalose-6-phosphate phosphatase pathway [27,28,29]. Compounds such as trehalose play a crucial role in the negative feedback regulation of TPS activity [30,31]. Evidence indicates that the capacity of TPS accumulation varies with temperature induction, as noted in the TPS levels of *Drosophila melanogaster* [32], *Delia antiqua* [33], *Maruca vitrata* [34], and *Megaphorura arctica* [35].

At present, some studies regarding *B. dorsalis* biology focus on constant and lethal temperatures [36,37,38,39], but the temperatures often deviate far from the normal temperature in natural environment. In order to identify the physiological responses and developmental parameters of oriental fruit fly to temperature stress, we detected the effects of various short-term high- and low-temperature treatments on the survival rate, fecundity, levels of trehalose, and other resistant substances in *B. dorsalis*. This study aims to provide a better understanding of tolerance strategy in the fly, and predict its development, survival, and reproduction in environments with different temperature stress to develop better management of the pest.

## 2. Materials and Methods

### 2.1. Test Insects

Larvae or adult *B. dorsalis* were collected in 2018 from infected kiwifruits in Shuicheng County, Guizhou, China (26°33′6.264″ N, 104°57′55.404″ E; alt. 1796 m). They were reared in a growth chamber (26 ± 1 °C, 70 ± 10% RH, and 14/10 h light/dark cycle). Colonies of *B. dorsalis* were regularly rejuvenated with wild ones. Adults were housed in screen cages (30 cm × 30 cm × 30 cm) and supplied with artificial food and water. Eggs and larvae were given a diet mixture with 7.2 g wheat bran, 8.4 g bean dregs, 10.8 g yeast, 14.4 g sugar, 73.2 mL H_2_O, and other micronutrient antibiotics [40]. Adult flies were cultured using 60 g sugar, 20 g yeast powder, 6 g agar powder, 5 g peptone, 0.6 g methyl 4-hydroxybenzoate, 0.5 g sorbic acid, and 500 mL H_2_O [41]. The mature larvae were placed in 3 or 4 cm thick loose, moist sand soil to pupate. Healthy adults at 3–6 d of emergence were provided with bananas to lay eggs. The bananas were pierced with a toothpick before placing in cages [42]. Females readily laid eggs in the holes created by the toothpick. The areas with eggs were then washed and collected in water. Fresh eggs, larvae, pupae, and adults were selected as test insects and were assigned to each of 5 temperature regimens.

### 2.2. Temperature Treatments and Materials

The experimental group were subjected to short-term high- and low-temperature treatments at 0 °C, 5 °C, 10 °C, 31 °C, and 36 °C, corresponding to the temperature fluctuations that are common in agroecosystems. After 12 h in temperature-controlled incubators set to one of the above temperatures, the flies were removed and placed in a climatic chamber (26 °C) for further processing and observation. The control group was subjected to a constant temperature of 26 °C.

A disposable plastic cup with a lid (top diameter: 4 cm, bottom diameter: 3 cm, and height: 3 cm) was used as the oviposition device, which was punctured evenly to make approximately 80 holes (<2 mm in diameter) from the bottom [39]. Next, banana pieces (approximately 3 cm^3^) were placed in egg collection cups for adult oviposition. The cups were covered tightly. The bananas were purchased from a supermarket, washed, and refrigerated at 4 °C.

### 2.3. Short-Term Temperature Stress on Egg, Larval, and Pupal Developments

One day old eggs were used for this experiment. With a fine brush, 20 fruit fly eggs were placed on glass dishes (d = 10 cm) containing a 1.5 cm thick artificial diet. Exposure in an incubator that set at one of the 5 temperature stresses mentioned above for 12 h, and then the egg containing dishes were transferred to a climate chamber (26 °C) for rearing the insects until they hatched. The development and survival of the eggs were observed under a stereoscopic microscope every 24 h until the eggs turned black or died. Four replicates were used per treatment.

For this study, 1-day-old larvae were captured and divided in groups of 20 larvae each. They were fed with an artificial diet of nearly 1.5 cm thickness placed on glass dishes. The larvae containing dishes were placed in the incubator that had been preset at a predetermined temperature for 12 h. The dishes were then moved to another chamber (26 °C) to continue rearing and ensuring adequate food supply. After the larvae attained maturity (exhibited bouncing behavior), they were placed in boxes (length: 17 cm, width: 11 cm, and height: 7 cm) filled with 3 or 4 cm sand. The larvae were observed under a stereoscopic microscope every day until pupation or death (larvae that died were counted as a straight body and no response to a mild stimulation with a fine brush was recorded). Each treatment was performed four times.

Next, 1-day-old pupae were divided in groups of 20 each and were placed on a glass dish lined with aqueous filter paper. The dishes were placed in a climatic chamber set at a predetermined temperature for 12 h. Then, they were transferred and allowed to incubate (26 °C). The moisture of filter paper was maintained until emergence. The development and survival of pupae were recorded every 24 h until all pupae emerged or died (black or dried-out pupae were considered dead). Each different temperature was considered a treatment, each treatment was repeated four times.

### 2.4. Short-Term Temperature Stress on the Survival, Longevity, and Fecundity of Adults

Adults were selected within 24 h of emergence. Each pair of male and female insects were placed in a rearing box (length: 17 cm, width: 11 cm, and height: 7 cm). Boxes containing a dish (3.5 cm in diameter) with a moistened cotton ball and a 1 cm^3^ artificial diet were subjected to temperature treatment for 12 h. After exposure to temperature stress, the adult insects were moved to 26 °C and supplied with fresh food. After 5 d of rearing, homemade oviposition cups containing fresh banana pieces were placed in the boxes to follow female insects’ oviposition. Food and oviposition cups were replaced every day. The preoviposition, reproduction, and longevity of adult insects were observed daily until the death of the last fly. Individual survival was recorded every day until 4 d. Eight pairs of male and female insects were used for each treatment, and four replicates were used per treatment.

### 2.5. Short-Term Temperature Stress on the Glucose, Glycogen, and Trehalose Levels of Adults

Glucose level was measured using the glucose Assay Kit (Suzhou Michy Biomedical Technology Co., Ltd., Suzhou, China). Adult flies subjected to various temperature treatments were separately weighed (0.1 g fresh body weigh) in 1.5 mL tubes. They were rapidly frozen in liquid nitrogen. Then, 1 mL of distilled water was added to the tubes, and the contents were homogenized using a freezing grinder, followed by soaking them for 10 min in a 95 °C water bath. After cooling, the homogenates were centrifuged at 8000× *g* for 10 min at room temperature. The supernatant was taken, and extracts were spiked following the manufacturer’s instructions. The absorbances of the obtained suspensions were recorded at 505 nm using a microplate reader. There were five replicates in each treatment.

Glycogen level was determined using the glycogen Reagent Kit (Suzhou Michy Biomedical Technology Co., Ltd., Suzhou, China). In brief, 0.1 g of adult insects was weighed in 1.5 mL tubes, which were rapidly frozen in liquid nitrogen. Next, 0.75 mL of extraction solution was added to the tubes. The contents were ground using a freezing grinder, then homogenates were transferred to 10 mL tubes and shaken once every 5 min for 20 min in a 95 °C water bath. After the tissues were dissolved, distilled water was added to the tubes to adjust the volume to 5 mL. Afterwards, the samples were cooled and stirred. The mixture was centrifuged at 8000× *g* for 10 min at 25 °C to obtain the supernatant. A sample of each tube was spiked in strict accordance with the manufacturer’s instructions. Absorbances were measured at 620 nm using a microplate reader. Each different temperature was considered a treatment, and five replicates were used per treatment.

Trehalose level was measured using a trehalose Reagent Kit (Suzhou Michy Biomedical Technology Co., Ltd., Suzhou, China). In brief, 0.1 g of adult insects subjected to various temperature treatments was rapidly frozen in liquid nitrogen and weighed in 1.5 mL Eppendorf tubes. Then, 1 mL of the extraction solution was added, the tube contents were thoroughly ground using a freezing grinder. After 45 min, the samples were left at room temperature and shaken 3–5 times. The samples were centrifuged at 10,000× *g* for 10 min at 25 °C, and the supernatant was used for further analysis. The sample was spiked in accordance with the manufacturer’s instructions. Absorbances were measured at 620 nm using a microplate reader. Each treatment was repeated five times.

### 2.6. Short-Term Temperature Stress on Adult TPS Activity

TPS activity was determined using the TPS Reagent Kit method (Suzhou Michy Biomedical Technology Co., Ltd., Suzhou, China). Samples were rapidly frozen in liquid nitrogen. Next, 1 mL of extraction solution was added, and then the mixture was ground using a freezing grinder. Samples were centrifuged at 8000× *g* for 10 min at 25 °C. The supernatant was chilled and subjected to TPS activity assessment in accordance with the kit manufacturer’s instructions. Absorbances were measured at 505 nm using a microplate reader. Five replicates (six adults per replicate) were used per treatment.

Protein level of the adult insects was measured via bicinchoninic acid method [43] with the Protein Assay Kit (Suzhou Kemin Biotechnology Co., Ltd., Suzhou, China). Absorbances were measured at 562 nm using a microplate reader.

### 2.7. Statistical Analysis

One-way analysis of variance (ANOVA) followed by Tukey’ s multiple range test (*p* < 0.05) using IBM SPSS Statistics 19.0 was used to analyze the developmental periods, sex ratio, preoviposition period, fecundity, and adult longevity data. Paired *t*-test was used to determine the difference in longevity between male and female at the same temperature [44]. Before performing ANOVA, the data were subjected to exploratory analyses to verify normality and homogeneity respectively using the Shapiro–Wilk and Levene’s tests. All values were expressed as means ± standard errors in the study. The percentage of sex ratio datasets was arcsine-transformed to improve normality and homoscedasticity. The bar graphs were created using Origin 2019 in the text.

The estimated survival rate was analyzed using log-rank (Mantel–Cox) test with Kaplan–Meier analysis (*p* < 0.05). The figures were created using GraphPad Prism 8. When the factors varied significantly from homogeneous distribution, paired tests were performed on preordered means [45].

## 3. Results

### 3.1. Effects of Short-Term Temperature Stress on Survival Rates

**Figure 1:**
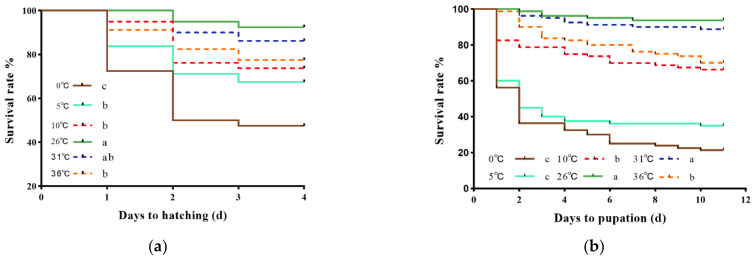

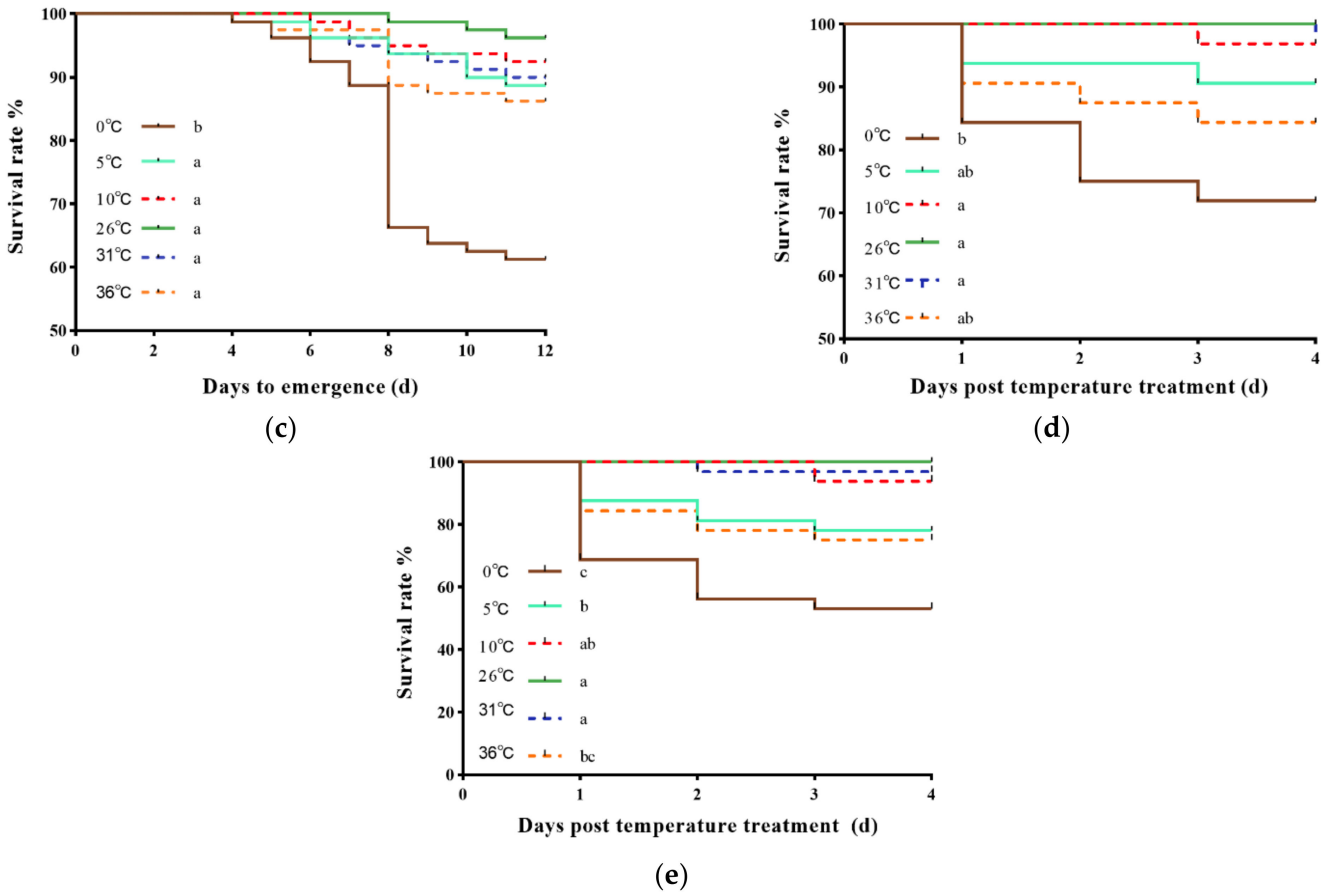
Survival rates of eggs (**a**), larvae (**b**), pupae (**c**), female adults (**d**), and male adults (**e**) of *B. dorsalis* subjected to various short-term high- and low-temperature treatments. The number of individuals analyzed in different stages: eggs = 80, larvae = 80, pupae = 80, female adults = 32, and male adults = 32. Different lowercase letters on the right side of the different groups indicate significant differences among different treatments. Survival rates were estimated using the Kaplan–Meier method (*p* < 0.05, log-rank (Mantel–Cox) test).

The survival rates of all stages of *B. dorsalis* were significantly affected by different short-term temperature treatments (Figure 1). After low temperature treatments, egg and larval stages had the lowest survival. When treated at 0 °C for 12 h, the survival rates of egg and larva were 47.50% at 3 d and 21.25% at 10 d, respectively. As the pupae and female adults were exposed to a short-term high-temperature treatment at 36 °C, their survival rates at 3 d still reached to 86.25% and 84.37%, respectively. In addition, the percent survival of male adults after exposure to 0 °C was significantly lower than that of the control, was 53.13% (χ^2^ = 9.021; *p* < 0.05). After temperature stress, the survival rate of all stages reached to the lowest at 0 °C. The survival rates of *B. dorsalis* with the above-mentioned treatments except pupae decreased sharply on the first day after treatments.

### 3.2. Effects of Short-Term Temperature Stress on Developmental Periods

As shown in Table 1, significant differences in the egg, larval, and pupal stages were noted after various temperature treatments. At low temperatures, both the egg and larval stages developed for significantly longer than the control group, with the egg stage prolonging by >2-fold at 0 °C. The development duration of eggs, larvae, and pupae were the shortest at 26 °C and prolonged after exposure to 0 °C, 5 °C and 36 °C. The developmental periods of pupae were significantly longer after exposure to ≤5 °C than after exposure to other temperatures. Overall, short-term temperature treatments exerted a considerable effect on the egg and larval stages compared with the effects on the pupal developmental period, implying that the pupae were more tolerant to temperature stress.

### 3.3. Effects of Short-Term Temperature Stress on the Female Ratio in the Pupae Emerged and Preoviposition Period

**Figure 2:**
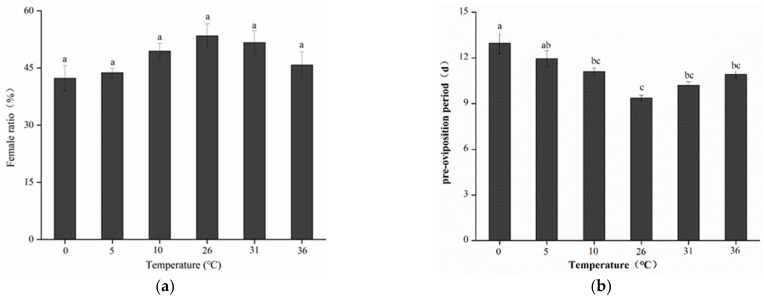
Female ratio in the pupae emerged (**a**) and preoviposition period (**b**) of adults after 12 h of high- and low-temperature treatments. Different lowercase letters denote significant differences among insects subjected to various temperature treatments (*p* < 0.05, Tukey’s test). Data are expressed as means ± standard errors of four replicates.

No significant differences were noted in the female ratio in the emerged pupae of *B. dorsalis* subjected to various short-term temperature treatments (Figure 2). In addition, the proportion of female in the pupae emerged gradually decreased as the temperature increased or decreased, reaching the lowest of 42.30 ± 3.33% at 0 °C.

The preoviposition duration of *B. dorsalis* was significantly affected after 12 h of exposure to various temperature treatments (F5,18 = 10.11; *p* < 0.05). Compared with that noted in the control group, preoviposition was significantly prolonged when the treatment temperature was 0 °C, or was 12.97 ± 0.69 d. Preoviposition duration was the shortest at 26 °C (9.37 ± 0.19 d). With the increase in temperature, the preoviposition period of *B. dorsalis* was first shortened and then prolonged. No significant difference was noted between control insects and those exposed to 36 °C (10.91 ± 0.23 d).

### 3.4. Effects of Short-Term Temperature Stress on Fecundity

**Figure 3:**
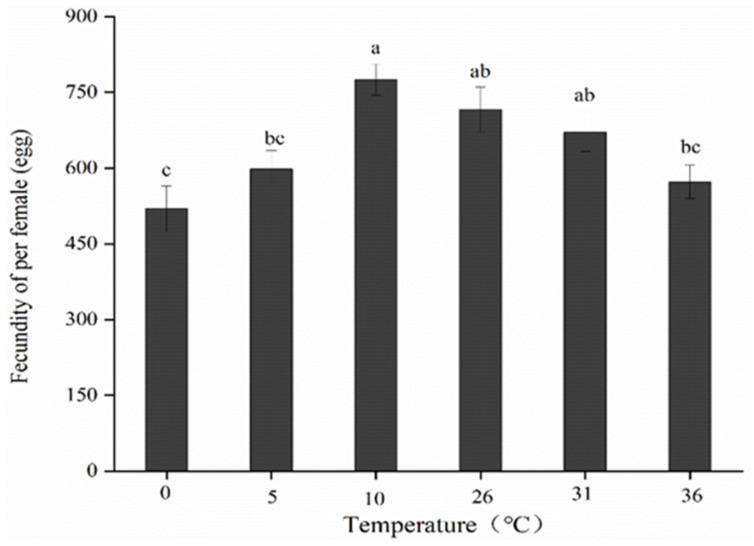
Fecundity of per female exposed to various short-term high- and low-temperature treatments. Different lowercase letters indicate significant differences among insects subjected to various temperature treatments (*p* < 0.05, Tukey’s test). Data are expressed as means ± standard errors of four replicates.

Subjecting adults to short-term high- and low-temperature treatments significantly affected their fecundity (F5,18 = 8.805; *p* < 0.05) (Figure 3). During the short-term high-temperature treatment, the egg number per female decreased gradually with increasing temperature. When the temperature was increased to 36 °C, the number of eggs laid by a female was significantly lower than that of the control group. Upon low-temperature treatments, the egg number per female decreased gradually. The maximum fecundity was noted at 10 °C (775.53 ± 31.10 eggs), which was slightly higher than that noted in the control group (716.05 ± 44.38 eggs). Egg production at 5 °C and 0 °C was altered significantly, the lowest number of eggs produced was 519.63 ± 40.70 at 0 °C.

### 3.5. Effects of Short-Term Temperature Stress on Longevity

**Figure 4:**
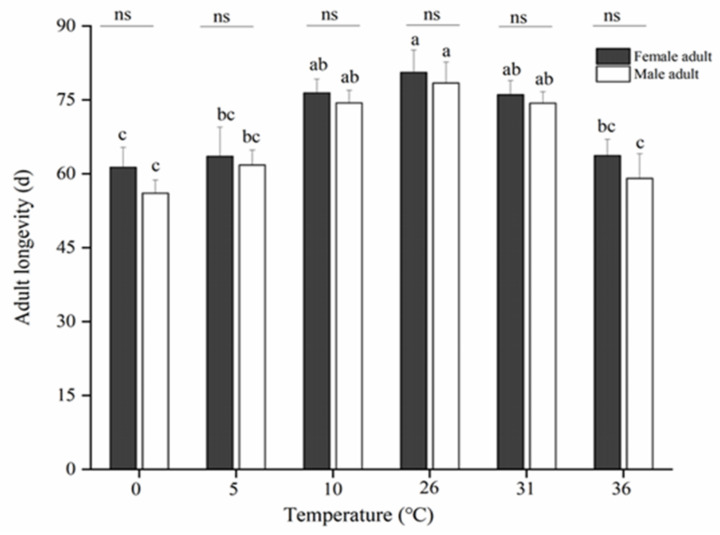
Adult longevity of *B. dorsalis* after 12 h of high- and low-temperature treatments. Different lowercase letters on the same color bar indicate significant differences (*p* < 0.05, Tukey’s test). Data are expressed as means ± standard errors of four replicates. ns (*p* > 0.05) indicates the longevities of females and males at same temperature are not significantly different.

Exposure to various short-term temperatures significantly affected the longevity of adults (Figure 4). When the treatment temperature was decreased to 0 °C (Female: 60.53 ± 3.33 d; Male: 57.00 ± 4.53 d) or increased to 36 °C (Female: 63.13 ± 2.83 d; Male: 59.60 ± 3.48 d), the longevity of female or male adults decreased gradually compared with that at 26 °C (Female: 80.20 ± 2.68 d; Male: 78.43 ± 3.69 d), suggesting that temperature stress shortens the lifespan of both sexes. Nonetheless, no significant difference was noted in longevity after exposure to 10 °C (Female: 76.33 ± 3.57 d; Male: 75.20 ± 3.92 d) and 31 °C (Female: 75.77 ± 2.97 d; Male: 75.36 ± 1.92 d). In general, the longevity of fruit flies decreased gradually with short-term temperature treatments.

### 3.6. Effects of Short-Term Temperature Stress on Low-Molecular-Weight Carbohydrate Levels

**Figure 5:**
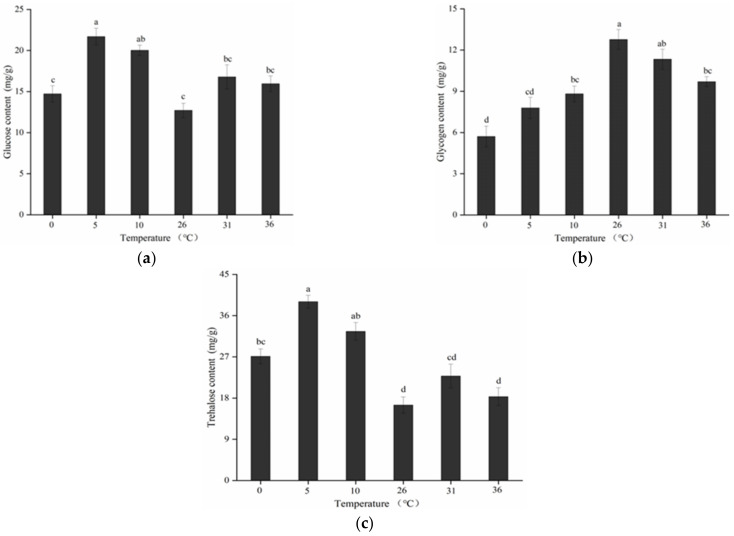
Glucose (**a**), glycogen (**b**), and trehalose (**c**) levels of adult *B. dorsalis* exposed to various short-term high- and low-temperature treatments. Data are presented as means ± standard errors of five replicates. Different lowercase letters indicate significant differences (*p* < 0.05, Tukey’s test).

As shown in Figure 5, significant differences in the levels of glucose (F5,24 = 10.757; *p* < 0.05), glycogen (F5,24 = 14.173; *p* < 0.05), and trehalose (F5,24 = 20.369; *p* < 0.05) were detected after various short-term temperature treatments. The glucose level increased from 12.71 ± 0.87 mg/g of fresh body weight at 26 °C to 21.70 ± 1.02 mg/g of fresh body weight at 5 °C. However, it decreased as the temperature dropped to 0 °C, no significant difference was noted between the results obtained at 0 °C and in the control group. The glucose level increased at high temperature, but the difference in glucose levels noted at 31 °C and 36 °C was not significant.

Compared to high and low temperatures, the glycogen level was the highest in the control group, where it was 12.77 ± 0.72 mg/g of fresh body weight. It was the lowest content at 0 °C with 5.71 ± 0.75 mg/g of fresh body weight. When the treatment temperature dropped to 0 °C, there was significant difference between the glycogen content of 0 °C and control group. Furthermore, the glycogen level at 36 °C was significantly lower than it was at the control temperature. Overall, as the temperature increased or decreased, the glycogen level decreased.

Trehalose was the major carbohydrate found in *B. dorsalis* adults under various short-term temperature stress conditions. The trehalose level was 16.47 ± 1.78 mg/g of fresh body weight in the control group. It increased linearly with decreasing temperature and peaked at approximately 39.06 ± 1.40 mg/g of fresh body weight at 5 °C. Afterwards, the level decreased slightly in response to temperature stress. The significant difference was noted in the results obtained at 0 °C and in the control group. Similar correlations were observed during high-temperature treatments.

### 3.7. Effects of Short-Term Temperature Stress on TPS Activity of Adults

**Figure 6:**
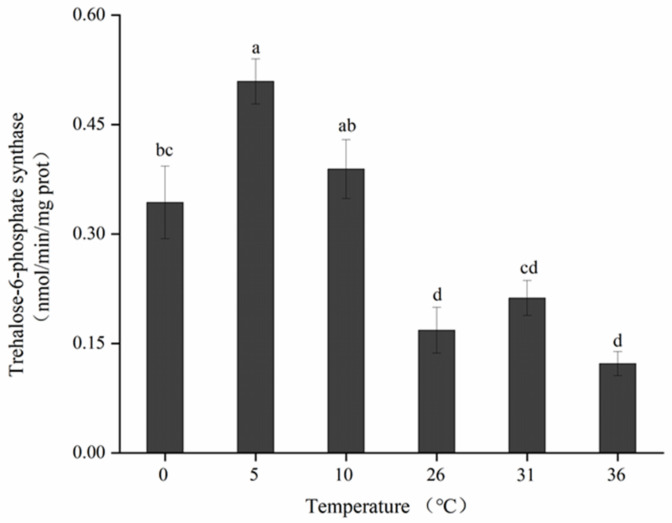
Trehalose-6-phosphate synthase activity of adult *B. dorsalis* exposed to various high- and low-temperature treatments. Different lowercase letters indicate significant differences (*p* < 0.05, Tukey’s test). Data are expressed as means ± standard errors of five replicates.

The activity of TPS in *B. dorsalis* was significantly dependent on short-term temperature treatment (Figure 6) (F5, 24 = 19.076; *p* < 0.05). Significant differences in TPS activity were observed at all low temperatures, and the highest activity of this enzyme was detected at 5 °C, which was 0.51 ± 0.02 nmol/min/mg prot. In contrast, the activity decreased as the temperature dropped <5 °C and increased by more than twice at 0 °C compared with that noted in the control group (0.17 ± 0.03 nmol/min/mg prot). TPS activity was altered in response to increases in the treatment temperature. The treatment at 36 °C resulted in an activity of 0.12 ± 0.02 nmol/min/mg prot, but the difference in TPS level (compared with the control group) was not significant. Furthermore, short-term high-temperature treatments exerted no effect on this enzyme’s activity in *B. dorsalis*.

## 4. Discussion

Insects exposed to heat or cold stresses may exhibit altered behavior, morphology, life history, and physiological characteristics, which negatively or positively affected their population [12,15,46]. In this study, we examined how short-term high- and low-temperature treatments affect the growth, development, fecundity, and sugar metabolism of *B. dorsalis*. In addition to having a harmful effect on phenology of the fly, our findings are indicative of *B. dorsalis* being more tolerant to high-temperature stresses compared to low-temperature stresses. The similar results have been showed in previous study [39,47]. This might be an explanation for its widespread damage in tropical and subtropical regions. In our experiment, the survival rates of the insect at all stages decreased gradually after short-term temperature treatments. It is worth noting that our results were not totally the same with the previous ones. For instance, as the temperature decreased or increased, the survival rates of female adults were at least 72%, which was higher than those of other developmental stages. This is similar to the finding that adults are more resilient [12,47], but the larvae stages are more tolerant due to the lower supercooling capacity and poor mobility [48,49,50]. These differences observed are common when working on the temperature stress response of insects from different instar, insect orders, or the experimental setup and insect treatment. For example, old instar larvae of the species were used by Manrakhan, while ours were newly produced within 24 h [48]. Several studies have reported a decreased survival rate of insects at various developmental stages under short-term temperature stress, including those of *B. carambolae* [51], *B. cucurbitae* [47], and *Ceratitis capitata* [13]. At 31 °C, no significant correlation was noted between the survival rates of all developmental stages and the control group. This may be because the optimal temperature for the growth and development of *B. dorsalis* is between 20 °C and 30 °C [39,48]. Although a suboptimal temperature of 31 °C exerted some detrimental effects on the insects, it did not act as a stressor. Furthermore, our results are consistent with the findings of Huang who reported that short-term temperature stress significantly prolongs the developmental periods of flies [47]. The chill coma temperature threshold is 5 °C for *B. dorsalis* [52], which may be exceeded, resulting in the cessation or slowing of insect development.

In the present study, adult insects were subjected to a short-term temperature treatment, which exerted a significant effect on their preoviposition period, fecundity, longevity, and sex ratio after pupal emergence. However, no significant correlation was identified in female ratio between the insect groups subjected to high- and low-temperature treatments. The overall male-to-female ratio showed a tendency to be nearly 1:1, and this ratio is largely determined by genes. The preoviposition period of *B. dorsalis* decreases with increasing temperature [3]. Our results support their conclusion—when the temperature exceeded the optimal temperature, preoviposition was delayed. In addition, decreasing temperature reduces longevity and fecundity, but a higher fecundity is noted in insects subjected to short-term treatments at moderately low temperatures [53]. The present study obtained similar findings in that the fecundity per female increased after a 12 h exposure to 10 °C followed by the constant temperature of 26 °C. The difference in reproduction may be because of the fact that 10.21 °C is the starting temperature for development [3], and treatment at 10 °C may contribute to the lab adaptation on adults. As evident from our results, the fecundity per female adult of *B. dorsalis* was reduced after various temperature treatments. We also found the one event temperature stress response of this fly was significantly different compared to their response of constant temperature stress, but both forms of high temperatures caused the low fecundity of the fly. For instance, thermal stress in *B. dorsalis* adults could still lay eggs of our experiment in a single event of 36 °C stress but at a constant temperature of 35 °C, no egg laying was observed for this species in a study conducted by Michel [39]. Insects often encounter unfavorable temperature environment, and they devote high levels of energy to metabolism, reproduction, and other life activities, which results in decreased adult longevity [54]. We observed that male adults had a shorter life span at 0 °C than female adults, indicating that the latter has a higher resistance than the former, and the shortest average life spans were 56.06 and 61.31 d for male and female adults, respectively. These results are inconsistent with the findings regarding the longevity of adults in the wild (a generation may survive for 26–61 (average: 39.2) d) [55]. This may be due to the more complex natural environment.

Insects follow environmental cues such as different temperatures to initiate adaptation mechanisms. Although temperature fluctuations within a certain range are beneficial for insects, unfavorable temperatures exert significant effects on the physiological and biochemical indicators of resistance and enzymatic responses in most insects [26,56]. The processes underlying temperature tolerance include the reduction of supercooling points and use of carbohydrate cryoprotectants such as sugar substances [14,57]. Our findings show that short-term high- and low-temperature treatments of *B. dorsalis* adults exhibit a strong relationship with glucose, glycogen, and trehalose levels. This suggests that the resistance of *B. dorsalis* to unfavorable temperatures is a complex process involving the coordinated action of multiple substances associated with stress resistance. Our studies on the levels of these carbohydrates revealed that the highest increases in glucose and trehalose levels occurred at 5 °C and the lowest increases occurred at 0 °C. Furthermore, because glycogen degrades into monosaccharides and sorbitol, a decrease in glycogen level may result in an increase in glucose and trehalose levels. The production of carbohydrates is most likely a response to the cue of extreme temperatures, as they act as anti-resistance agents [17]. In general, the trehalose level increased initially but decreased later in response to the various temperature treatments. One possible explanation is that trehalose that accumulates in adult *B. dorsalis* and converts into glucose via the decomposition function of trehalose as protective substances, which plays a crucial role in exposing this pest to temperature stress [30,58]. This may explain why *B. dorsalis* has a lower survival rate at extreme temperatures, and energy is diverted from normal life activities to resist the temperature stress, which impairs insect fitness and even survival. This finding is consistent with previous findings that trehalose is a major component for energy storage and its pattern of utilization varies across insects subjected to various temperature treatments [14,21,32]. However, *Gampsocleis gratiosa* subjected to various temperature treatments for 24 h and 48 h exhibited changes in its trehalose level [58]. This indicates that the duration of exposure affects the trehalose level.

Changes in the activity of insects’ protective enzymes are considered a significant indicator of their stress tolerance [29,59]. The accumulation of trehalose has been associated with the induction of TPS in certain insects under extreme environmental stresses [20,22,28]. As demonstrated by our results, short-term high- and low-temperature treatments resulted in significant increases in TPS activity and enhanced activity at both low temperatures and 31 °C. Previous studies have established a relationship between TPS and temperature stress [32,33,34,35]. The present study demonstrated that exposure to various temperature treatments for 12 h significantly affected the levels of glucose, glycogen, and trehalose, as well as the activity of TPS in *B. dorsalis*, but whether the treatment duration exceeded their maximum level for resistance adaptation remains to be determined.

In conclusion, treatments at extremely short-term temperatures not only affected the survival of eggs and larvae most, but also exerted detrimental impacts on development, adult longevity, and fecundity of *B. dorsalis*. We confirmed that temperature stress is a major potential contributor to generate trehalose metabolism substances. Such low-molecular-weight carbohydrates and TPS activity may contribute to temperature tolerance. These findings are crucial for predicting the effects of climate change on this pest’s population growth and resistance to heat and cold stresses. In this study, however, we provided adults access to food and water prior to and after treatment, although this might be more limiting in natural environment and subject to a large number of interacting variables. This can pose a challenge to our experimental results. Therefore, similar research conducted under natural conditions would help to provide further knowledge of the population dynamics of the spices. In addition, to control *B. dorsalis* infestation more effectively, additional studies on fruit fly’s trehalose metabolism in response to temperature stress are warranted.

## Figures and Tables

**Table 1:** Developmental periods of different stages of *B. dorsalis* subjected to various short-term high- and low-temperature treatments.

Temperature (°C)	Developmental Period (d)		
	Egg	Larva	Pupa
26	1.56 ± 0.07 ^d^	8.15 ± 0.16 ^d^	10.06 ± 0.03 ^b^
0	3.11 ± 0.17 ^a^	10.11 ± 0.34 ^a^	11.25 ± 0.16 ^a^
5	2.68 ± 0.05 ^b^	9.68 ± 0.06 ^ab^	11.08 ± 0.03 ^a^
10	2.35 ± 0.14 ^bc^	9.39 ± 0.13 ^ab^	10.28 ± 0.07 ^b^
31	1.82 ± 0.04 ^d^	8.43 ± 0.07 ^cd^	10.01 ± 0.14 ^b^
36	2.23 ± 0.07 ^c^	9.19 ± 0.08 ^bc^	10.25 ± 0.07 ^b^

Data are expressed as means ± standard errors of four biological replicates. Different lowercase letters in each column (stage) denote significant difference in the developmental periods of different stages of the same insect (*p* < 0.05, Tukey’s test).

## Data Availability

Data are available upon request from the authors.
